# Effects of detraining after blood flow-restricted low-load elastic band training on muscle size and arterial stiffness in older women

**DOI:** 10.1186/s40064-015-1132-2

**Published:** 2015-07-15

**Authors:** Tomohiro Yasuda, Kazuya Fukumura, Haruko Iida, Toshiaki Nakajima

**Affiliations:** Department of Ischemic Circulatory Physiology, Graduate School of Medicine, University of Tokyo, Tokyo, Japan; School of Nursing, Seirei Christopher University, 3453, Mikatahara, Kita-ku, Hamamatsu, Shizuoka 433-8558 Japan

**Keywords:** Muscle hypertrophy, Blood flow restriction, Elastic-band, Biceps brachii, Exercise

## Abstract

**Background:**

We examined the effects of detraining after blood flow-restricted (BFR) low-load elastic band training on muscle size and arterial stiffness in older women.

**Findings:**

Fourteen women were divided into BFR training (BFR-T) or non-BFR training (CON-T). Each group participated in 12 weeks of arm curl and press down training using an elastic band either with (BFR-T) or without BFR (CON-T). Muscle cross-sectional area (CSA) and maximum voluntary isometric contraction (MVIC) for upper arms and cardio-ankle vascular index (CAVI) were evaluated before and after the 12-week training period and also after 12 weeks of detraining. CSA and MVIC were higher at post and detraining (CSA: 16.3% (p < 0.01) and 6.9% (p < 0.01) for elbow flexion and 17.1% (p < 0.01) and 8.7% (p > 0.05) for elbow extension; MVIC: 7.3 and 3.9% (both p > 0.05) for elbow flexion and 17.6 and 15.1% (both p < 0.01) for elbow extension) than at pre for the BFR-T, but not for the CON-T. There was no change in CAVI for the two groups.

**Conclusions:**

Increased muscle strength/size following 12 weeks of elastic band BFR-T was well maintained with a low risk of arterial stiffness after 12 weeks of detraining in older women.

## Background

Age-related skeletal muscle loss (sarcopenia) inhibits mobility and increases the risk of falls, fractures, disability, and heart disease (Haykowsky et al. 2013; Visser et al. 2005). Traditional high-load resistance training (HRT, ≥70% 1-repetition maximum: 1-RM) with weight machines/free weights produces favorable effects on skeletal muscle morphology and function in young and older adults (American College of Sports Medicine 2009). Therefore, HRT is an effective countermeasure to sarcopenia. In addition, it is reported that after the cessation of training (detraining) for the same length of time as the training period (12 weeks), muscle hypertrophy/strength responses are decreased but they still retain higher levels than the pre-training level (Tokmakidis et al. 2009; Kubo et al. 2010).

HRT cannot be performed in elderly patients with abnormalities of the bone or joint (i.e., multiple sclerosis patients, hip/knee arthritis patients). However, resistance training of as low as 20–30% 1-RM with weight machines/free weights combined with blood flow restriction (BFR-T) results in changes in muscle hypertrophy and strength comparable to those observed with HRT, regardless of age (Takarada et al. 2000; Karabulut et al. 2010). In addition, Yasuda et al. (2014c) revealed that muscle size and strength are increased following BFR-T when exercises are performed at low-resistance levels using an elastic band, as well as when using free weights. Elastic bands are inexpensive, compact, and easy to use compared with machines/free weights, making elastic band BFR-T an affordable and easy to use home-based resistance training program to serve as a countermeasure to attenuate muscle atrophy with aging (Mikesky et al. 1994; Colado and Triplett 2008). Although it has been demonstrated that the increased muscle size/strength obtained by BFR-T using machines or free weights is preserved for the detraining period (Yasuda et al. 2014c, 2015), it is unclear whether muscle adaptation responses with elastic band BFR-T can be retained following detraining.

HRT reduces central arterial compliance, which may be associated with high rates of mortality in patients with end-stage renal failure and essential hypertension (Miyachi et al. 2004). However, these values return to the baseline levels during the detraining period (Miyachi et al. 2003, 2004), suggesting that the effect of resistance training on the cardiovascular system is different between training and detraining periods. In contrast, BFR-T does not render negative effects on arterial stiffness (Ozaki et al. 2013; Yasuda et al. 2014b). However, the effect of detraining after BFR-T on the cardiovascular system is currently unknown. Thus, we investigated the effects of detraining after elastic band BFR on muscle size, muscle strength and arterial stiffness in older women. A previous study (Wernbom et al. 2007) reviewed that the average length of the training period was approximately 3 months (11-13 weeks) for dynamic resistance training. In the present study, therefore, training and detraining periods of 12 weeks each were used.

## Methods

**Figure 1:**
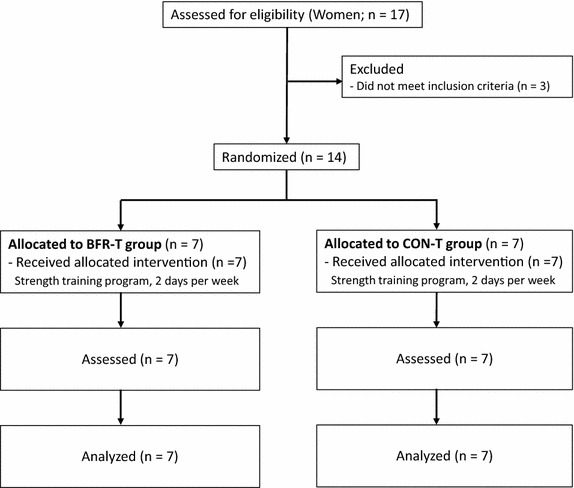
Consolidated standards of reporting trials (CONSORT) diagram.

Fourteen untrained Japanese older women (aged 61–85 years) volunteered to participate in this study (Figure 1). All participants qualified based on the exclusion criteria proposed by Greig et al. (1994) (blood pressure >160/100 mmHg, body mass index >30 kg/m^2^, history of anemia, cerebrovascular disease, myocardial infarction and arthroscopic joint surgery) used to define “medically stable” older participants for exercise studies. In addition, any participant who suffered from a chronic disease, such as severe hypertension (>180/110 mmHg), orthopedic disorders, deep venous thrombosis, peripheral vascular disease, or cognitive dysfunction were excluded from the study. All participants were free of overt chronic disease as assessed by medical history, physical examination, and complete chemistry and hematologic evaluation. Eight participants were classified as “recreationally active”; 6 women participated in regular aerobic-type exercise (walking or jogging; 2–4 times/week for approximately 30–60 min) and 2 women had light to moderate resistance training experience and performed lower body training, but they were not professionally trained. Others (6 women) were classified as “sedentary”. All participants were randomly divided into BFR-T (n = 7, mean ± SD: age 72 ± 7 years, standing height 1.55 ± 0.06 m) or non-BFR training (CON-T) (n = 7, mean ± SD: age 67 ± 6 years, standing height 1.54 ± 0.06 m). BFR-T and CON-T groups performed bilateral arm curl and triceps press down exercise training 2 days/week for 12 weeks. Both exercise groups used a “Thin (Yellow)” band (Hygenic Corporation; Akron, Ohio, USA). This training was performed under the close supervision of those with technical knowledge in BFR training. Training volume was 75 repetitions (30, 15, 15 and 15 reps, with 30 s rests between sets) for both exercises (90 s rests between exercises). This protocol is typical of submaximal BFR studies. Once the pneumatic cuffs were inflated, they remained inflated for the two exercises, including the rest periods between sets and exercises. During arm curl exercise, participants were comfortably seated on a chair (Yasuda et al. 2014a). Elbow joint range of motion (ROM) during the exercise was approximately 20–145° (0° being full extension). During the triceps press down exercise, participants were comfortably seated on a rowing chair with the body supported in the vertical position (Yasuda et al. 2014a). Elbow joint ROM during the exercise was approximately 140–5° (0° being full extension). Participants were instructed not to allow the band to snap back to the start position, but rather to consciously control the return movement such that it would take twice as long as the stretching movement. The repetition duration was 2.4 s (1.2-s concentric and 1.2-s eccentric exercise cycle) for both exercises. The BFR-T wore a specially designed pneumatic cuff (30-mm width, KAATSU Master, KAATSU Japan Co., Ltd., Tokyo, Japan) around the most proximal portion of the both arms. On the first day of training, the cuffs were set at 30 mmHg and gradually inflated to 120 mmHg (Day 1). The training air pressure was increased by 10–20 mmHg at each subsequent training session until a pressure of approximately 270 mmHg was reached if the subject could perform at high levels of pressure intensity. The mean pressure intensity throughout the period of training was 202 ± 8 mmHg (230–270 mmHg at 24th training session). The cuffs were inflated to the target pressure for the entire exercise session including rest periods between sets and exercises. The total length of the two exercises per day was 9.5 min for both groups. Both groups were informed of the risks associated with the methods and procedures and signed an informed consent document before participation. During the detraining period (12 weeks), participants stopped resistance training, and returned to their normal daily activities as prior to the resistance training period. There was no change (p > 0.05) in body mass for BFR-T (pre, 47.0 ± 6.2; post, 46.7 ± 6.6; detraining, 46.8 ± 7.0 kg) and CON-T (pre, 50.6 ± 5.6; post, 49.9 ± 5.2; detraining, 50.0 ± 5.2 kg).

To determine the relative exercise loading of performing the arm curl and triceps press down exercises, the integrated EMG (iEMG) for the elastic band exercises (three to five repetitions) were normalized to maximum voluntary isometric contraction (MVIC) of the elbow flexors or elbow extensors. This measurement was completed on the same day before the 23rd training session. The coefficient variation for this measurement from test to retest was 5.7% (Yasuda et al. 2014c).

MVIC of the elbow flexors and extensors were measured by a dynamometer (Taiyo Kogyo Co., Tokyo, Japan). Each participant was comfortably seated on an adjustable chair, with the arm positioned on a stable table at chest level with the elbow bent at an angle of 90° (0° at full extension). The upper arm was maintained in the horizontal plane (at 90°), while the wrist was fixed at the end of the dynamometer lever arm in a position of supination for elbow flexion and in a position halfway between supination and pronation for elbow extension. Both forces were measured with a transducer while the participants performed two trials separated by a 60-s rest interval (90-s rests between elbow flexion and elbow extension). If MVIC torque for the first two MVICs varied by more than 5%, up to two additional MVICs were performed with 60-s rest between trials. Participants were instructed to perform an MVIC as quick as possible during a period of about 2 s. The recorded value for the MVIC was taken as the highest and most stable approximately 1 s of the 2-second contraction. The highest iEMG value during MVIC was used for data analysis [as a normalized EMG (% MVIC)]. The coefficient variation for this measurement from test to retest was 1.3%. The intraclass correlation coefficient of the measurements was 0.97 (Yasuda et al. 2014c).

Muscle cross-sectional area (CSA) was obtained using an MRI scanner (0.2-Tesla, Hitachi, Tokyo, Japan). A T-1 weighted, spin-echo, axial plane sequence was performed with a 500-ms repetition time and a 23-ms echo time. Participants rested quietly in the magnet bore in a supine position, with their arms extended along their trunk. Continuous transverse images with 10-mm slice thickness were obtained from both upper arms of the body. All MRI data were transferred to a personal computer for analysis using software (sliceOmatic, Tomovision Inc., Montreal, QC, Canada). Muscle CSA was measured for elbow flexors and extensors at 6 cm and 16 cm above the elbow joint, respectively (Yasuda et al. 2014c). The coefficient variation of this measurement was less than 1.0%.

Flow-mediated dilatation (FMD) of the brachial artery was measured using an instrument equipped with software for monitoring the brachial artery diameter (UNEX EF, Unex Co. Ltd., Nagoya, Japan) (Yeboah et al. 2009). Cardio-ankle vascular index testing (CAVI) and ankle brachial pressure index (ABI) were measured noninvasively using a VS-1500 system (Fukuda Denshi Co., Ltd., Tokyo, Japan) (Shirai et al. 2006). Venous blood samples (5 mL) were obtained from the antecubital vein for coagulation system (fibrin/fibrinogen degradation products [FDP] and D-dimer), indirect marker/indicator of muscle damage [creatine kinase (CK)] and oxidative stress [derivatives of reactive oxygen metabolites (dROM) and biological antioxidant potential (BAP)]. The levels of dROM and BAP were measured using a free radical elective evaluator (Free carpe diem, Diacron International, Grosseto, Italy) (Kanaoka et al. 2010). All measurements were completed before (Pre) and within 3–7 days after (Post) the training, and also 12 weeks after the training period (Detraining).

### Statistical analyses

Results are expressed as mean ± SD. The data were tested for normality using Shapiro–Wilk test. Because all variables were normally distributed, parametric statistical analyses were performed. Two-way ANOVA with repeated measures [condition (BFR-T, CON-T) by time (pre, post) detraining] was used to evaluate the training effects for all dependent variables. When significant main effects or interaction were observed, post hoc testing was performed using Tukey post hoc test. Statistical significance was set at p < 0.05. Pre/Post and post/detraining effect sizes (ESs, Cohen’s *d*) for MVIC, muscle CSA, hemodynamic parameters, arterial function coagulation system, muscle damage and oxidative stress were calculated with the following formula: [post mean − pre mean]/pre SD, [detraining mean − pre mean]/post SD; *d* < 0.2 is a trivial effect, *d* = 0.2–0.5 is a small effect, *d* = 0.5–0.8 is a moderate effect, and *d* > 0.8 is a large effect (Cohen 1988).

## Results

Before training, there were no significant differences between two groups for age (p = 0.181), anthropometric variables (standing height, p = 0.692; body weight, p = 0.272), MVIC (elbow flexion, p = 0.419; elbow extension, p = 0.991), muscle CSA (elbow flexors, p = 0.330; elbow extensors, p = 0.679) (Table 1), hemodynamic parameter and arterial function (p = 0.150–0.448), coagulation system, muscle damage and oxidative stress (p = 0.435–1.000) (Table 2). In BFR-T and CON-T groups, there were no differences (p > 0.05) for normalized EMG (arm curl: 26 and 30% MVIC, press down: 33 and 31% MVIC). During BFR-T and CON-T exercises, no participant performed contraction efforts until exhaustion. During both exercises, no sign of discomfort or pain was observed in the participants.

**Table 1:** Changes in MVIC and muscle CSA after 12 weeks of training and detraining periods

	BFR-T (n = 7)					CON-T (n = 7)				
	Pre	Post	DT	Post ES	DT ES	Pre	Post	DT	Post ES	DT ES
MVIC										
Elbow flexion (EF), N	125 (20)	135 (22)^§^	130 (17)	0.45^a^	0.20^a^	118 (15)	115 (19)	118 (20)	−0.19	0.00
Elbow extension (EE), N	91 (25)	106 (27)**	104 (28)*	0.61^b^	0.50^b^	91 (17)	92 (14)	89 (13)	0.06	−0.14
Muscle CSA										
EF muscle CSA (cm^2^)	11.6 (2.0)	13.4 (1.9)**	12.4 (3.8)**^,¶¶^	0.94^c^	0.41^a^	10.8 (1.1)	10.7 (0.9)	11.3 (1.0)	−0.04	0.49^a^
EE muscle CSA (cm^2^)	12.4 (2.1)	14.3 (1.3)**	13.4 (1.9)	0.89^b^	0.47^a^	12.0 (1.6)	12.0 (1.4)	11.6 (1.6)	0.03	−0.25

Values are mean (SD). *ES* effect size, *DT* detraining. *Post ES* Pre to Post ES, *DT ES* *Pre to detraining ES, MVC* maximal voluntary isometric strength, *CSA* cross-sectional area, *BFR-T* low-intensity resistance training with blood flow restriction and *CON-T* low-intensity resistance training without blood flow restriction.

** vs. pre, p < 0.01. * vs. pre, p < 0.05. ^§^vs. pre, p = 0.13. ^¶¶^ vs. post, p < 0.01.

^a^Small ES.

^b^Moderate ES.

^c^ Large ES.

**Table 2:** Changes in hemodynamic parameter, arterial function coagulation system, muscle damage and oxidative stress after 12 weeks of training and detraining periods

	BFR-T (n = 7)			CON-T (n = 7)		
	Pre	Post	Detraining	Pre	Post	Detraining
Hemodynamic parameter and arterial function						
Heart rate (bpm)	69 (21)	68 (10)	68 (12)	62 (10)	59 (5)	64 (7)
Systolic BP (mmHg)	140 (16)	139 (19)	143 (20)	126 (18)	120 (10)	122 (8)
Diastolic BP (mmHg)	82 (11)	83 (10)	83 (13)	79 (11)	77 (9)	79 (7)
FMD (%)	3.5 (2.0)	3.7 (2.1)	4.6 (2.2)	4.3 (1.8)	3.9 (1.8)	4.3 (1.4)
CAVI (m/s)	9.1 (1.3)	9.3 (1.1)	9.0 (1.1)	8.4 (0.8)	8.2 (0.8)	8.3 (0.6)
ABI (unit)	1.14 (0.06)	1.12 (0.08)	1.11 (0.08)	1.08 (0.09)	1.07 (0.06)	0.96 (0.36)
Coagulation system, muscle damage and oxidative stress						
FDP	2.9 (0.9)	3.3 (1.0)	3.4 (0.8)	2.9 (0.4)	3.0 (0.8)	3.1 (1.5)
D-dimer	0.21 (0.08)	0.25 (0.11)	0.26 (0.15)	0.25 (0.12)	0.25 (0.20)	0.27 (0.22)
CK	109 (52)	107 (43)	107 (24)	94 (19)	90 (41)	94 (43)
d-ROM	369 (60)	351 (68)	367 (81)	396 (63)	372 (76)	377 (65)
BAP	1,863 (279)	2,091 (367)	2,101 (146)	1,964 (324)	2,197 (154)	1,985 (248)

Values are mean (SD). *BFR-T* low-intensity resistance training with blood flow restriction, *CON-T* low-intensity resistance training without blood flow restriction, *BP* blood pressure, *FMD* flow-mediated dilatation, *CAVI* cardio-ankle vascular index testing, *ABI* ankle brachial pressure index, *FDP* fibrin/fibrinogen degradation products, *CK* creatine kinase, *dROM* derivatives of reactive oxygen metabolites and *BAP* biological antioxidant potential.

A condition by time interaction was observed in the MVIC for elbow extension (p = 0.016) and in the muscle CSA for elbow flexion (p < 0.001) and extension (p = 0.035), and tended to be observed in the MVIC for elbow flexion (p = 0.061). MVIC were higher and tended to be higher at post-training (p = 0.128 for elbow flexion and p = 0.006 for elbow extension) and detraining (p = 0.027 for elbow extension) than at pre-training for the BFR-T, but not for the CON-T. Muscle CSA was higher at post-training (p < 0.001 for elbow flexion and p = 0.009 for elbow extension) and detraining (p = 0.009 for elbow flexion) than at pre-training for the BFR-T, but not for the CON-T (Table 1). There were no changes (p > 0.05) in hemodynamic parameters, arterial functions, coagulation system, muscle damage and oxidative stress for both groups over the duration of the experiment (Table 2).

The magnitude of changes in muscle size and strength between pre- and post-training was always larger for BFR-T (small, moderate or large) than that for CON-T (trivial). For BFR-T, the ESs for muscle size from pre to detraining were small, and the ESs from pre to detraining for muscle strength were small or moderate. For CON-T, the ESs for muscle size and strength from pre to detraining were trivial (Table 1). After training and detraining periods with BFR-T, the rate of average change in muscle CSA was 1.36 and −0.78% per week for elbow flexors, and 1.42 and −0.70% per week for elbow extensors, respectively. The rate of average change in MVIC was 0.61 and −0.29% per week for elbow flexion, and 1.46 and −0.20% per week for elbow extension, respectively.

## Discussion

As with previous HRT studies (12 weeks training and 12 weeks of detraining) (Tokmakidis et al. 2009; Kubo et al. 2010), our findings in older women indicate that 12 weeks of detraining after 12 weeks of elastic band BFR-T decreased the muscle strength/size, but higher levels than pre-training were still retained. In addition, we observed no changes in arterial function after detraining as well as during the training period.

In this study, the increased arm muscle CSAs following 12 weeks training with elastic band BFR-T were decreased at 12 weeks detraining but they retained higher levels (small effect) than pre-training level (Table 1). This result was similar to those in a previously reported HRT study (Tokmakidis et al. 2009) in that increased muscle size following HRT was retained for the same period of detraining in older adults. In fact, the ratio of change (training and detraining periods) for muscle CSA was similar between BFR-T for elbow flexion (1.36 vs. −0.78% per week, respectively) and elbow extension (1.42 vs. −0.70% per week, respectively) and the previously reported HRT study (0.80 vs. −0.53% per week, respectively). A previous study has demonstrated that protein synthesis (i.e., mechanistic target of rapamycin signaling pathway) was increased during BFR-T (Fry et al. 2010), but protein degradation (i.e., myostatin) is reported to be associated during training/detraining with BFR-T as well as HRT (Laurentino et al. 2012; Schiaffino et al. 2013). Taken together, it appears that protein degradation (myostatin and mTOR signaling pathway) is involved for older women during the period of detraining with BFR-T using elastic bands as well as machines.

In this study, increased MVIC for elbow extension following the training also remained elevated above the pre-training level after detraining (Table 1). This result was similar to those of previously reported HRT studies (Fatouros et al. 2005; Tokmakidis et al. 2009) in that increased muscle strength following HRT was retained for the same period of detraining in older adults. On the other hand, the increase in muscle strength for elbow flexion tended to be higher (p = 0.13) only after the 12 weeks of training (Table 1). As in the present study, a previous study (Matta et al. 2011) demonstrated that increased MVIC following 12 weeks of training (24 sessions) was lower in elbow flexion (9.4%) compared with elbow extension (13.4%). It can be speculated that elbow extension achieved greater muscle strength during the training period, so there were statistical differences in MVIC following training and detraining between the two muscle groups. On the other hand, there have been no studies to compare the effects of BFR training/detraining period on muscle adaptation among muscle groups. Therefore, it should be noted that a potential mechanism for time course of muscle strength might differ among the muscle groups. Further study is necessary to better understand the relationship between muscle strength and each muscle group following BFR training/detraining period.

HRT (4 months) negatively affects arterial functions, but the reduced arterial function returns to the baseline level after 2–4 months of detraining (Miyachi et al. 2004). In contrast, our results demonstrated that hemodynamic parameters, arterial stiffness, vascular endothelial function, coagulation factors, muscle damage and oxidant stress did not change over the duration of the experiment. Recently, Ozaki et al. (2013) demonstrated that carotid arterial compliance decreased in HRT but not in BFR-T, and the changes were correlated with systolic blood pressure elevations during exercise sessions. In addition, unlike HRT, the effects of elastic band training on hemodynamic parameters were not largely affected (Zion et al. 2003). Consequently, it appears that elastic band BFR-T did not have a negative effect on the cardiovascular system after detraining as well as during training.

The present study has some limitations. First, it should be noted that our sample size was small. In this study, the effect size in MVIC (0.45–0.61) and muscle CSA (0.89–0.94) following the training period was not high; they were associated with the low-to-moderate level of effect size (0.20–0.50 and 0.41–0.47, respectively) following the detraining period. Thus, future studies employing a more robust experimental design with a large sample size should be undertaken to verify the finding in this study. Second, there was no non-training control group in this study. Therefore, future studies with a more extensive experimental design and a large sample size consisting of three (BFR-T, CON-T, and no exercise) groups should be undertaken to verify the findings in this study. Third, since the cuff pressure intensity for arms was higher than that reported in the previous studies (Ozaki et al. 2013; Yasuda et al. 2015), it is necessary to pay close attention to the side effects of BFR-T. Fourth, we did not assess the details of habitual physical activities, body flexibility and endocrine resistance training/detraining.

In conclusion, increased arm muscle strength and size following 12 weeks of elastic band BFR-T were decreased at 12 weeks of detraining but higher levels were retained compared to the pre-training level, and arterial function did not change over the duration of the experiment. Thus, a home-based resistance training program using elastic band BFR-RT could serve as a practical and effective means of preventing/improving sarcopenia in older women.

## Abbreviations

1-RM: one repetition maximum
BFR-T: blood flow restricted low-load resistance training
HRT: high-load resistance training
MVIC: maximum voluntary isometric contraction
NBFR-T: no-blood flow restricted low-load resistance training
